# A YOLO Ensemble Framework for Detection of Barrett’s Esophagus Lesions in Endoscopic Images

**DOI:** 10.3390/diagnostics15182290

**Published:** 2025-09-10

**Authors:** Wan-Chih Lin, Chi-Chih Wang, Ming-Chang Tsai, Chao-Yen Huang, Chun-Che Lin, Ming-Hseng Tseng

**Affiliations:** 1 Master Program in Medical Informatics, Chung Shan Medical University, Taichung 40201, Taiwan; s0958041@gm.csmu.edu.tw; 2 Division of Gastroenterology and Hepatology, Department of Internal Medicine, Chung Shan Medical University Hospital, Taichung 40201, Taiwan; cshy1192@csh.org.tw (C.-C.W.); tsaimc1110@gmail.com (M.-C.T.); forest65@csmu.edu.tw (C.-C.L.); 3 School of Medicine, Chung Shan Medical University, Taichung 40201, Taiwan; moongenius0913@gmail.com; 4 Institute of Medicine, Chung Shan Medical University, Taichung 40201, Taiwan; 5 Information Technology Office, Chung Shan Medical University Hospital, Taichung 40201, Taiwan

**Keywords:** object detection, YOLO ensemble, Barrett’s Esophagus, healthcare

## Abstract

**Background and Objectives**: Barrett’s Esophagus (BE) is a precursor to esophageal adenocarcinoma, and early detection is essential to reduce cancer risk. This study aims to develop a YOLO-based ensemble framework to improve the automated detection of BE-associated mucosal lesions on endoscopic images. **Methods**: A dataset of 3620 annotated endoscopic images was collected from 132 patients. Five YOLO variants, YOLOv5, YOLOv9, YOLOv10, YOLOv11, and YOLOv12, were selected based on their architectural diversity and detection capabilities. Each model was trained individually, and their outputs were integrated using a Non-Maximum Suppression (NMS)-based ensemble strategy. Multiple ensemble configurations were evaluated to assess the impact of fusion depth on detection performance. **Results**: The ensemble models consistently outperformed individual YOLO variants in recall, the primary evaluation metric. The entire five-model ensemble achieved the highest recall (0.974), significantly reducing missed lesion detections. Statistical analysis using McNemar’s test and bootstrap confidence intervals confirmed the superiority in most comparisons. **Conclusions**: The proposed YOLO ensemble framework demonstrates enhanced sensitivity and robustness in detecting BE lesions. Its integration into clinical workflows can improve early diagnosis and reduce diagnostic workload, offering a promising tool for computer-aided screening in gastroenterology.

## 1. Introduction

Barrett’s esophagus (BE), first described by British surgeon Norman Barrett in the 1950s, is histologically characterized by the replacement of the stratified squamous epithelium in the distal esophagus with specialized columnar epithelium [1]. This pathological change is closely associated with gastroesophageal reflux disease (GERD) and is widely recognized as the primary precursor to esophageal adenocarcinoma (EAC) [2,3]. However, due to the often asymptomatic nature of BE and the current diagnostic reliance on endoscopic examination and histopathological biopsy, early detection remains particularly challenging [4]. Clinically, diagnosis is based on the endoscopic identification of tongue-like projections or salmon-colored mucosa in the distal esophagus, with biopsy confirmation of intestinal metaplasia. Notably, the prevalence of BE is rising in Asia [5], and given the generally poor five-year survival rate for EAC, typically below 20% [6], effective surveillance and early diagnosis of BE are of significant clinical value for delaying or preventing malignant transformation.

Recent advances in artificial intelligence (AI) have introduced new opportunities for automated analysis and decision support in medical imaging. Deep learning architectures, particularly convolutional neural networks (CNNs), have achieved remarkable performance in image classification and visual recognition tasks. Since the success of AlexNet in the ImageNet competition [7], models such as VGGNet, ResNet, and Inception have further advanced the semantic representation capabilities of computer vision [8,9].

In the field of object detection, deep learning has replaced traditional hand-crafted feature engineering with end-to-end architectures capable of learning high-level semantic features directly from data. Frameworks such as the R-CNN family [10] and YOLO (You Only Look Once) [11] have significantly improved detection accuracy and processing speed and are now widely adopted in domains, including surveillance, autonomous driving, and medical image analysis. Recent studies have also demonstrated the utility of deep learning in the interpretation of endoscopic images to detect BE and associated abnormalities [12,13]. For example, Yu et al. [14] proposed a multi-task deep learning model that integrates classification, image retrieval, and segmentation modules to assist endoscopists in identifying esophageal cancer and esophagitis. Tsai et al. [15] developed an AI system based on EfficientNetV2B2 that achieved a accuracy of 94.37% in the diagnosis of BE, highlighting its clinical diagnostic potential. AI systems with real-time image processing capabilities can provide dynamic interpretive suggestions during endoscopic procedures, thus facilitating earlier recognition of lesions, guiding targeted biopsies, and potentially reducing unnecessary sampling, thus optimizing clinical resource allocation and patient management [16].

More recent AI papers that focus specifically on esophageal lesion detection in endoscopic images include the following: Jukema et al. [17] showed that AI-supported CADx workload could improve the characterization performance of general endoscopists during BE assessment to expert levels. Aoyama et al. [18] studied early esophageal squamous cell carcinoma (ESCC) using AI on endoscopic videos, demonstrating improved sensitivity and accuracy when AI assistance is combined with endoscopist readings, boosting diagnosis from around 57.4% to 66.5% sensitivity and 68.6% to 75.9% accuracy. Baik et al.’s [19] research used multicenter data with white light imaging (WLI) and narrow band imaging (NBI) trained on the YOLOv5 and RetinaNet models, achieving up to 98.4% accuracy and 91.3% sensitivity in NBI for esophageal lesion detection, showing strong performance under various imaging conditions. Li et al. [20] reviewed and studied exploring AI applications for Barrett’s esophagus and related lesion detection reveal AI systems that achieve detection accuracy rates greater than 83% to 90%, enhancing the ability to localize areas in real time during endoscopy. Chen et al. [21] used multicenter randomized controlled trials applying AI models such as YOLOv5 directly integrated within endoscopy equipment, which reduced missed rates and provided real-time lesion identification with sensitivity close to 90%, employing methods such as heat maps for lesion visualization.

These recent studies have showcased cutting-edge advances in artificial intelligence for esophageal lesion detection using endoscopic imagery, with particular emphasis on early cancer identification, enhanced diagnostic accuracy, and real-time clinical deployment. These works underscore the expanding role of deep learning in improving diagnostic accuracy and minimizing missed lesions. However, most existing methodologies rely on single-model architectures, which may limit robustness and generalizability. In contrast, the present study proposes a novel ensemble framework that integrates multiple variants of YOLO, in order to improve detection sensitivity and reliability in the identification of Barrett’s esophageal lesions.

The YOLO family of models has emerged as a pivotal advance in object detection. Its end-to-end design allows for simultaneous bounding-box regression and classification in a single inference pass, greatly improving efficiency and processing speed [11]. Since the release of YOLOv1, the architecture has undergone successive refinements (Table 1): YOLOv2 introduced multi-scale training and dimension clustering; YOLOv3 integrated spatial pyramid pooling (SPP) and Darknet-53 for enhanced feature extraction; YOLOv4 adopted CSPDarknet and the Mish activation function to improve accuracy. Subsequent iterations, YOLOv5 to YOLOv7, shifted to anchor-free mechanisms, optimized parameter efficiency via E-ELAN, and introduced Transformer components to increase generalization. Recent versions such as YOLOv8–v12 have integrated cutting-edge innovations including generative adversarial networks (GANs), panoptic segmentation, FlashAttention, and depth-wise separable convolutions, further advancing detection performance and broadening application scope.

Ensemble learning is a technique aimed at improving predictive performance by integrating multiple base learners. Its core philosophy is that combining predictions from diverse learners can mitigate variance, bias, or overfitting issues, thus enhancing model stability and accuracy. While individual YOLO models are known for their efficiency and precision, they may still experience missed detections or misclassifications when faced with complex or highly variable data. To address this, recent studies have incorporated ensemble strategies to aggregate outputs from multiple models and bolster system robustness. Numerous works have validated the effectiveness of ensemble learning in object detection tasks. For example, Liu et al. combined YOLOv5 models of various input sizes for railway surveillance, achieving an accuracy of 85.4% [22]; Singh proposed the YORES model, integrating YOLO with ResNet to improve vehicle classification [23]; Hu et al. fused YOLOv6, YOLOv7, and Faster R-CNN to reach an F1 score of 90.5% in crowd surveillance [24]; and Lv combined EfficientDet with YOLOv8 for thermal vehicle detection, outperforming standalone models [25]. Collectively, these studies affirm that both homogeneous and heterogeneous ensemble approaches offer promising improvements in detection accuracy and stability.

**Table 1:** Evolution of YOLO Architecture [26].

Release	Year	Tasks	Contributions	Framework
Yolo [11]	2015	Object Detection, Basic Classification	Single-stage object detector	Darknet
Yolov2 [27]	2016	Object Detection, Enhanced Classification	Multi-scale training, dimension clustering	Darknet
Yolov3 [28]	2018	Object Detection, Multi-scale	SPP block, Darknet-53 backbone	Darknet
Yolov4 [29]	2020	Object Detection, Basic Tracking	Mish activation, CSPDarknet-53 backbone	Darknet
Yolov5 [30]	2020	Object Detection, Instance Segmentation	Anchor-free detection, SWISH activation, PANet	PyTorch
Yolov6 [31]	2022	Object Detection, Instance Segmentation	Self-attention, anchor-free OD	PyTorch
Yolov7 [32]	2022	Object Detection, Tracking, Segmentation	Transformers, E-ELAN reparameterization	PyTorch
Yolov8 [33]	2023	Object Detection, Instance and Panoptic Segmentation	GANs, anchor-free detection	PyTorch
Yolov9 [34]	2024	Object Detection, Instance Segmentation	PGI and GELAN	PyTorch
Yolov10 [35]	2024	Object Detection	Consistent dual assignments for NMS-free training	PyTorch
Yolov11 [36]	2024	Object Detection, Instance Segmentation	Expanded capabilities, improved efficiency	PyTorch
Yolov12 [26]	2025	Object Detection, Instance Segmentation	Advanced attention-centric design (Flash Attention, R-ELAN), 7 × 7 separable convolutions, improved computational efficiency	PyTorch

This study aims to investigate the feasibility and performance of applying YOLO models for the detection of mucosal lesion regions in Barrett’s esophagus endoscopic imagery, with a particular focus on ensemble methods to improve robustness and reliability. The specific objectives are as follows:

- To analyze the lesion detection and localization performance of individual YOLO models on BE image datasets, using established object detection metrics.
- To compare performance between different versions of YOLO and evaluate their respective strengths in feature extraction and recognition of lesions, identifying the most suitable architecture for BE analysis.
- To further enhance detection performance, ensemble learning techniques are applied to integrate the output of various YOLO variants. This multi-model fusion strategy is designed to reduce model-specific detection inconsistencies, thereby strengthening the robustness and adaptability of lesion localization across heterogeneous image presentations.

The contributions of the study include the design of a novel ensemble framework using five YOLO variants, a clinically aligned evaluation strategy prioritizing recall, a progressive fusion analysis of ensemble depth, and rigorous statistical validation. Collectively, these elements demonstrate the originality and its potential impact on AI-assisted endoscopic diagnosis.

## 2. Materials and Methods

**Figure 1:**
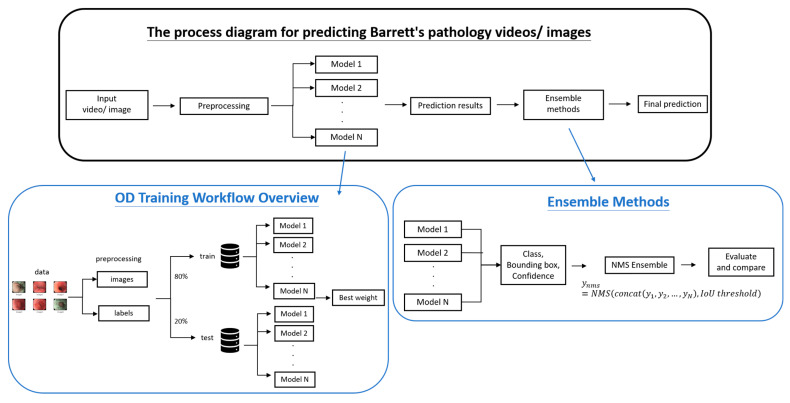
Research Workflow Diagram.

This study leverages YOLO-based object detection models, enhanced through ensemble learning strategies, to improve the performance and robustness of mucosal lesion detection in Barrett’s esophagus (BE) imagery. As shown in Figure 1, the workflow comprises four major phases: data preprocessing, YOLO model training, ensemble integration, and performance evaluation.

### 2.1. Data Preprocessing

Input images were first preprocessed through annotation and resizing to enhance model generalizability during training. The dataset was collected from Chung Shan Medical University between March 2023 and March 2025 and comprises endoscopic video recordings from 132 patients, 97 diagnosed with BE and 35 non-BE controls. This study used de-identified clinical images and videos collected from hospital records.

Still frames were extracted from continuous endoscopic footage and experienced clinicians selected representative images to ensure clarity and diagnostic relevance. While this approach was intended to improve annotation quality and model training stability, we acknowledge that selecting only high-quality frames may introduce bias and potentially inflate performance metrics. The dataset includes a range of lesion morphologies, but future work will aim to incorporate more diverse image quality and lesion stages to better reflect real-world clinical variability and reduce selection bias.

**Figure 2:**
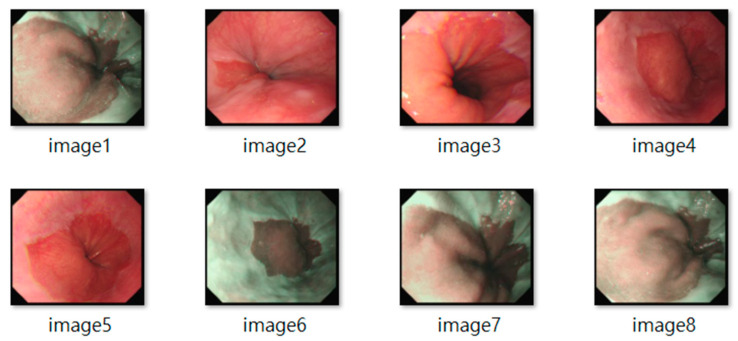
Representative Endoscopic Images of Barrett’s Esophagus (BE).

**Figure 3:**
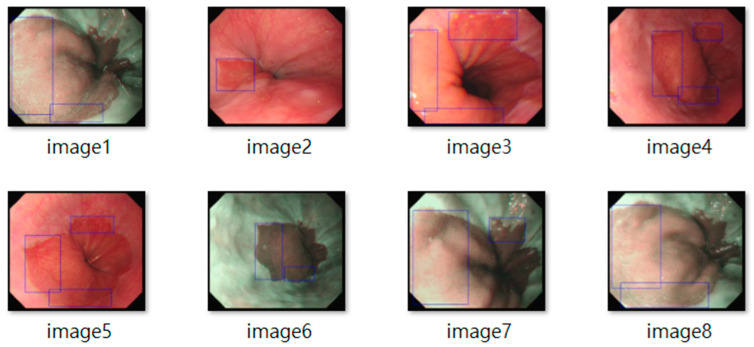
Expert-Annotated Lesion Regions in BE Endoscopic Imagery.

All frames underwent standardization (e.g., resolution normalization) and encompassed both white-light imaging (RGB) and narrow-band imaging (NBI) modalities. Representative samples are shown in Figure 2. The resulting dataset included 3620 labeled images, with 2220 from BE patients and 1400 from non-BE patients. All patient data were anonymized to comply with ethical standards. Lesion annotations were performed by expert endoscopists, with precise delineation of abnormal regions as shown in Figure 3.

### 2.2. YOLO Model Training

Five versions of the YOLO model, namely YOLOv5, YOLOv9, YOLOv10, YOLOv11 and YOLOv12, were selected for comparative evaluation in BE image analysis. These models were chosen due to their proven capabilities in real-time detection tasks and their iterative improvements in feature extraction, computational efficiency, and generalization. Although YOLOv6, YOLOv7, and YOLOv8 are widely used in object detection tasks, they were excluded from this study due to architectural redundancy with the selected models or limited performance improvements observed in preliminary trials on our dataset. A brief architectural summary is provided below.

- YOLOv5: The first PyTorch-based implementation, featuring an anchor-free architecture and PANet-based feature aggregation.
- YOLOv9: Incorporate PGI and GELAN modules to enhance feature extraction in complex scenarios.
- YOLOv10: Refines training strategies to improve bounding box consistency and stability.
- YOLOv11: Focuses on computational efficiency and architectural flexibility for broader deployment.
- YOLOv12: The latest release, which integrates Flash Attention, R-ELAN, and 7 × 7 depthwise separable convolutions in depth for lightweight attention-guided learning.

All data were divided into training and testing sets in an 8:2 ratio. Uniform training parameters were applied:

- Learning rate: 0.001, adjusted dynamically to accelerate convergence and prevent overfitting.
- Batch size: 16, balancing computational efficiency and stability.
- Epochs: 500, based on performance convergence trends.
- Optimizer: Adam, suitable for nonlinearities and sparse gradients.

Hyperparameter values were selected based on a combination of default settings provided by the YOLO framework and the previous experience with similar medical imaging tasks. Training was carried out on an NVIDIA GeForce RTX 4090 GPU (Micro-Star INT’L Co., Ltd., New Taipei, Taiwan), with data augmentation (random rotation, scaling, flipping) and early stopping employed to improve model robustness and generalization.

### 2.3. YOLO Ensemble Learning Strategy

To enhance the performance and stability of Barrett’s Esophagus (BE) image analysis, this study adopts an ensemble learning approach that integrates the predictions of five YOLO models: YOLOv5, YOLOv9, YOLOv10, YOLOv11, and YOLOv12. By combining the output of multiple models, the ensemble strategy mitigates biases and variances inherent in individual models, thus strengthening the detection capability for BE lesion regions.

The ensemble fusion strategy in this study was implemented using a Non-Maximum Suppression (NMS) based approach. For each image, predictions from all selected YOLO models were aggregated, and overlapping bounding boxes were refined by evaluating confidence scores and Intersection over Union (IoU) thresholds. This process eliminates redundant and low-confidence detections, improving overall precision and decision quality. No explicit score averaging or weighting was applied; instead, standard NMS was used to retain the most confident and non-overlapping predictions. The ensemble configurations (dual, triple, quadruple, and full model) were designed to assess the impact of architectural diversity and fusion depth on detection performance. While this approach provided a straightforward integration mechanism, we acknowledge that alternative fusion strategies such as weighted voting, soft NMS, or attention-based fusion may offer further improvements and will be considered in future work.

Based on preliminary performance assessments of five individual YOLO models, a stepwise ensemble integration strategy was employed to systematically improve detection sensitivity. At each stage of the ensemble construction, the model that contributed the most substantial improvement in recall was selectively incorporated. This approach ensured that each addition to the ensemble was based on its ability to expand lesion coverage and reduce false negatives. Through this progressively layered ensemble design, the fused outputs were evaluated using NMS. Comparative analysis was conducted to determine the effect of stepwise ensemble strategies on the performance of BE image detection.

### 2.4. Model Evaluation

To comprehensively assess the performance of YOLO-based ensemble learning methods for BE image analysis, this study used three primary evaluation metrics. Precision, recall and F1-score. Recall was prioritized as the main performance indicator, reflecting the clinical importance of maximizing lesion detection sensitivity in early screening contexts.

- Precision quantifies the proportion of correctly identified region of lesions among all regions predicted as lesions, reflecting the accuracy of the model predictions.
- Recall measures sensitivity by evaluating its ability to successfully detect the actual lesion regions.
- F1-score, the harmonic mean of precision and recall, offers a balanced evaluation metric particularly suited for imbalanced class scenarios.

The metrics are formally defined by the following equations:

(1) $$Precision=\frac{TP}{TP+FP}$$

(2) $$Recall=\frac{TP}{TP+FN}$$

(3) $$F1=\frac{2\times Precision\times Recall}{Precision+Recall}$$

where *TP* (True Positive) denotes correctly identified lesion regions, *FP* (False Positive) refers to incorrectly predicted non-lesion regions, and *FN* (False Negative) indicates missed lesions.

Given the paired nature of the detection results across models, we applied McNemar’s test to statistically evaluate the differences in recall between the complete ensemble model and each individual YOLO variant. The McNemar test assesses whether the proportion of discordant classifications between two models is significantly different, which is appropriate for binary paired outcomes (lesion detected vs. not detected).

For robustness, we also computed bootstrap confidence intervals (95% CI) for the difference in recall between each ensemble–individual model pair. We used 1000 stratified bootstrap resamples of the test dataset to capture sampling variability.

To maintain continuity with our original analysis, the P-score values (as defined in Equation (4) in the initial submission) are reported alongside the McNemar *p*-values in a supplementary column for reference. The *p*-values reported from McNemar’s tests are not adjusted for multiple comparisons and should be interpreted with caution due to the increased risk of false positives.

## 3. Results

This section presents a comprehensive evaluation of the detection performance of individual YOLO models and their ensemble counterparts on Barrett’s Esophagus (BE) endoscopic images. The findings are organized into three parts: (1) performance of individual YOLO models, (2) performance of ensemble models, and (3) comparative analysis between the two.

### 3.1. Performance of Individual YOLO Models

To compare the detection capabilities of five YOLO variants, we first investigated how intersection over union (IoU) thresholds and confidence thresholds influenced detection outcomes. Initial parameter tuning was performed using YOLOv12n, the latest model, under various configurations: IoU thresholds of 0.5 and 0.3, and confidence thresholds of 0.25 and 0.1. Table 2 presents the results for YOLOv12n across these settings, evaluated using precision, recall, and F1-score.

When the IoU was set to 0.3 and the confidence threshold to 0.25, YOLOv12n achieved its highest F1-score (0.879), with corresponding precision and recall values of 0.874 and 0.884, respectively. Lowering the confidence threshold further to 0.1 increased the recall to 0.922, indicating a greater sensitivity to potential lesions, but led to a drop in precision to 0.782, suggesting a modest increase in false positives.

Given the clinical goal of early detection and minimizing missed diagnoses, recall was prioritized over precision in this study. Therefore, the optimal parameter setting of IoU = 0.3 and confidence threshold = 0.1 was selected for subsequent evaluations. These settings were uniformly applied to the remaining YOLO models (YOLOv5n, YOLOv9t, YOLOv10n, and YOLOv11n) to assess architectural performance differences under consistent conditions.

Table 3 summarizes the performance of all five YOLO variants using the selected configuration. In general, each individual model demonstrated competent performance in detecting BE lesion. YOLOv11n achieved the highest recall (0.946), indicating strong sensitivity in identifying lesion areas, which is crucial for reducing false negatives in clinical screening. Meanwhile, YOLOv5n and YOLOv12n offered balanced performance across precision and F1-score, suggesting stability and better generalizability. YOLOv9t and YOLOv10n, while not leading in any specific metric, maintained consistent and reliable detection performance. These complementary strengths across models offer a compelling rationale for ensemble-based integration.

### 3.2. Performance of YOLO Ensemble Models

To further enhance the detection performance of Barrett’s Esophagus (BE) images, this study conducted a stepwise ensemble strategy, in which a YOLO model was incrementally added at each stage according to its contribution to maximizing recall. The evaluation results of these ensemble configurations, specifically in terms of precision, recall, and F1-score, are summarized in Table 4.

According to the outcomes presented in Table 4, increasing the number of models in the ensemble introduces a noticeable trade-off among the performance metrics. The dual-model ensemble of YOLOv11n and YOLOv9t achieved the highest precision (0.743) along with a balanced recall (0.957), suggesting a favorable balance between detection accuracy and lesion coverage. When incorporating YOLOv5n into a three-model ensemble, recall improved significantly to 0.968. However, precision decreased to 0.686 and the F1-score decreased to 0.807, indicating that the broadened detection scope may have introduced more false positives. Subsequent integration of YOLOv12n in the four-model ensemble and the final inclusion of YOLOv10n in the five-model ensemble further increased the recall to 0.971 and 0.974, respectively. Nevertheless, these enhancements in sensitivity were accompanied by gradual reductions in precision (to 0.676 and 0.667), along with slight decreases in F1-score, reflecting a trade-off where gains in sensitivity came at the expense of specificity. Overall, the results demonstrate a clear trend: As the ensemble size increases, recall improves consistently, while precision and F1-score tend to diminish.

### 3.3. Comparative Analysis of Individual vs. Ensemble Models

In this study, recall was selected as the primary performance evaluation metric, as it effectively reflects the sensitivity in identifying the regions of the lesion. In the context of medical image analysis, high recall indicates the model’s ability to capture as many potential lesions as possible, which is critical to reducing missed diagnoses and improving diagnostic performance. Accordingly, recall serves as the core criterion for performance assessment throughout this research.

We conducted a comparative analysis of recall performance between individual YOLO models and various ensemble configurations in the task of detecting Barrett’s Esophagus (BE) lesions, with the results summarized in Table 5. The findings demonstrate that the best-performing full ensemble model consistently outperforms any single model in terms of recall, further validating the effectiveness of the ensemble learning strategy in enhancing model sensitivity. By aggregating the predictions from multiple YOLO architectures, the ensemble approach substantially reduces the likelihood of missed detections, particularly in cases involving heterogeneous or poorly defined lesion boundaries. This results in more robust and reliable recognition performance.

To further assess the statistical significance of recall improvements, we applied the P-score method as described in Roiger (2017) [37]. This metric was originally proposed in the data mining literature and is used to compare error rates between two models. In our context, the error rate is defined as *E* = 1 − *recall*, where *E*_1_ and *E*_2_ represent the error rates of the ensemble model and the individual YOLO model, respectively. The average error rate is calculated as *q* = (*E*_1_ + *E*_2_*)*/2, and the sample size is denoted by *n*. The P-score formula is:

(4) $$P_{S}=\frac{\left|E_{1}-E_{2}\right|}{\sqrt{q\left(1-q\right)\left(\frac{1}{n_{1}}+\frac{1}{n_{2}}\right)}}$$

A P-score ≥ 2 indicates a statistically significant difference at the 95% confidence level. While this method is less commonly used in medical AI, we included it to complement McNemar’s test and bootstrap confidence intervals, providing an additional perspective on model comparison. For transparency and reproducibility, all variables used in the formula are defined and computed based on recall values and sample sizes from the test dataset.

To evaluate the statistical significance of recall improvements between the ensemble model and individual YOLO variants, we employed three complementary methods: 95 percent bootstrap confidence intervals (CI), McNemar’s test, and the P-score metric, as shown in Table 6. The bootstrap CI provides a non-parametric estimate of the variability in recall differences, while McNemar’s test assesses paired classification outcomes for each lesion detection. For YOLOv5n, YOLOv9t, YOLOv10n, and YOLOv12n, all three methods consistently indicated significant improvements. In contrast, YOLOv11n did not reach significance across any of the three tests. These results demonstrate internal consistency and reinforce the reliability of the ensemble model’s advantage in most comparisons. While the P-score is less commonly used in medical AI, it was included to complement standard methods and provide an additional perspective on performance evaluation.

## 4. Discussion

This study leverages the YOLO family of models to enhance the performance and stability of detecting Barrett’s Esophagus (BE) mucosal lesions through an ensemble learning strategy. Given the clinical objective of early warning and screening support, recall was deliberately selected as the main performance metric, as it reflects the sensitivity and comprehensiveness in identifying the regions of the lesion. In clinical contexts, the consequences of missed diagnoses typically outweigh those of false positives, making recall a more suitable indicator of the practical utility of automated diagnostic systems.

The proposed ensemble configuration, comprising YOLOv5n, YOLOv9t, YOLOv10n, YOLOv11n, and YOLOv12n, achieved a higher recall rate compared to any individual model, with statistically significant differences in performance. This highlights the strong potential in automated BE lesion detection tasks for early warning applications. Its implementation in future clinical decision support systems could substantially improve early screening and diagnostic sensitivity for BE. Meanwhile, in clinical endoscopic screening, FPR values below approximately 10–15% are typically considered acceptable if they contribute to improved sensitivity for early lesion detection. The full ensemble’s operating point in this study slightly exceeds this range, which can be explained by

1. Increasing the decision threshold to improve precision.
2. Using a secondary filtering classifier for low-confidence detections.
3. Implementing a two-stage review process to preserve recall while reducing false positives.

Although the ensemble model demonstrated consistent improvements in recall, this came at the cost of reduced precision and F1-score. Notably, the full ensemble achieved the highest recall (0.974) but also exhibited the lowest precision (0.667) and F1-score (0.792), indicating a substantial increase in false positive detections. In clinical settings, this trade-off must be carefully considered, as excessive false positives may lead to unnecessary biopsies, patient anxiety, and increased workload for healthcare providers. While high recall is essential for early lesion detection, maintaining diagnostic specificity is equally important to ensure efficient and accurate decision-making. To mitigate these effects, we propose several strategies: adjusting confidence thresholds to balance sensitivity and specificity, applying secondary filtering classifiers to reduce low-confidence detections, and implementing a two-stage review process to preserve high recall while improving precision. These approaches can help optimize the clinical utility of the ensemble framework in real-world applications.

However, several limitations warrant consideration. First, the dataset was sourced from a single medical institution, which may limit the model’s adaptability and generalizability across diverse clinical environments. Second, although the ensemble strategy improved recall, it was accompanied by a slight decline in precision in certain combination models, indicating room for further optimization. Meanwhile, future work may incorporate weighted ensemble mechanisms, attention-based modules, or uncertainty estimation to achieve a better balance between detection sensitivity and specificity. Additionally, this study focused solely on static image analysis. Extending the approach to real-time endoscopic video streams could improve their responsiveness and clinical practicality.

## 5. Conclusions

This study developed a robust ensemble learning framework using the YOLO model series (YOLOv5 to YOLOv12) to automate the detection of mucosal lesions associated with Barrett’s Esophagus (BE). By combining multiple YOLO variants, the system significantly enhanced recall, F1-score, and overall performance stability, demonstrating high sensitivity and suitability for clinical screening. Among the individual models, YOLOv11 achieved the highest recall of 0.946, showcasing strong lesion localization capabilities and serving as a solid base for ensemble strategies. All ensemble configurations outperformed individual models, with the full ensemble reaching a peak recall of 0.974. Statistical analysis confirmed these improvements as significant at the 95% confidence level, validating the ensemble approach’s effectiveness in boosting diagnostic sensitivity. The framework also exhibited strong robustness, consistency, and generalizability, making it highly promising for clinical integration. By effectively reducing the chances of missed pathological findings, the system provides clinicians with a reliable decision-support tool, enhancing early detection and prognosis of BE.

Future research may explore several directions to enhance the effectiveness and applicability of lesion detection models. These include multi-center validation to ensure the model performs reliably across diverse clinical sites and extending lesion detection capabilities from static images to live video for real-time performance evaluation. Integrating the model into endoscopic platforms could enable live lesion detection and assist with biopsy procedures. Additionally, developing intuitive user interfaces would help clinicians seamlessly incorporate the system into their diagnostic workflows. Finally, combining imaging data with clinical and genetic information through multimodal fusion could improve diagnostic accuracy and support personalized treatment planning.

In summary, the YOLO-based ensemble framework proposed in this study offers a feasible and effective technical solution for the automated diagnosis of BE. It has great potential to serve as a core component of future clinical decision support systems, further advancing the frontiers of intelligent healthcare.

## Figures and Tables

**Table 2:** Evaluation of Model Performance Across Parameter Settings.

Parameter	Precision	Recall	F1-Score
IoU = 0.5, confidence = 0.25	0.814	0.824	0.819
IoU = 0.3, confidence = 0.25	0.874	0.884	0.879
IoU = 0.3, confidence = 0.1	0.782	0.922	0.846

**Table 3:** Performance Results of Individual YOLO Models.

Model	Precision	Recall	F1-Score
Yolov5n	0.796	0.926	0.856
Yolov9t	0.755	0.934	0.835
Yolov10n	0.760	0.923	0.833
Yolov11n	0.717	0.946	0.816
Yolov12n	0.782	0.922	0.846

**Table 4:** Performance Results of YOLO Ensemble Models.

Ensemble Model	Precision	Recall	F1-Score
11n, 9t	0.743	0.957	0.836
11n, 9t, 5n	0.686	0.968	0.807
11n, 9t, 5n, 12n	0.676	0.971	0.799
11n, 9t, 5n, 12n, 10n	0.667	0.974	0.792

**Table 5:** Comparative Recall Performance between Single Models and Ensembles.

Yolo Model	Recall	Ensemble Model	Recall
Yolov5n	0.926	5n, 9t, 10n, 11n, 12n	0.974
Yolov9t	0.934		
Yolov10n	0.923		
Yolov11n	0.946		
Yolov12n	0.922		

**Table 6:** Statistical Comparison of Recall between Ensemble and Individual YOLO Models.

Ensemble Model	YOLO Model	Recall Diff	95% CI (Bootstrap)	McNemar’s Test *p*-Value	$P_{S}$ Value
5n,9t,10n,11n,12n	YOLOv5n	+0.048	0.026–0.072	0.004	2.953
	YOLOv9t	+0.040	0.018–0.065	0.009	2.582
	YOLOv10n	+0.051	0.029–0.077	0.003	3.095
	YOLOv11n	+0.028	−0.002–0.056	0.071	1.935
	YOLOv12n	+0.052	0.030–0.076	0.002	3.165

## Data Availability

The raw data supporting the conclusions of this article will be made available by the authors on request.
